# Dimethyl 2-(4-methyl­benzyl­idene)malonate

**DOI:** 10.1107/S1600536813012464

**Published:** 2013-05-18

**Authors:** Assem Barakat, Abdullah Mohammed Al-Majid, Yahia Nasser Mabkhot, M. Iqbal Choudhary, Sammer Yousuf

**Affiliations:** a Department of Chemistry, College of Science, King Saud University, PO Box 2455, Riyadh 11451, Saudi Arabia; b Department of Chemistry, Faculty of Science, Alexandria University, PO Box 426, Ibrahimia 21321 Alexandria, Egypt; c H.E.J. Research Institute of Chemistry, International Center for Chemical and Biological Sciences, University of Karachi, Karachi 75270, Pakistan

## Abstract

In the mol­ecule of the title compound, C_13_H_14_O_4_, the benzene ring forms dihedral angles of 18.60 (7) and 81.36 (8)° with the two arms of the malonate moiety. The crystal structure features C—H⋯O inter­actions, which form chains running parallel to the *b* axis.

## Related literature

For the biological activity and synthesis of alkyl­idene and aryl­idene malonates, see: Liu *et al.* (2012 ▶); Heydri & Tahamipour (2011 ▶); Xu & Wang (2011 ▶); Li *et al.* (2010 ▶, 2011 ▶); Gallier *et al.* (2009 ▶); Besavaiah *et al.* (2004 ▶). For the structures of related compounds, see: Rappoport & Gazit (1986 ▶)
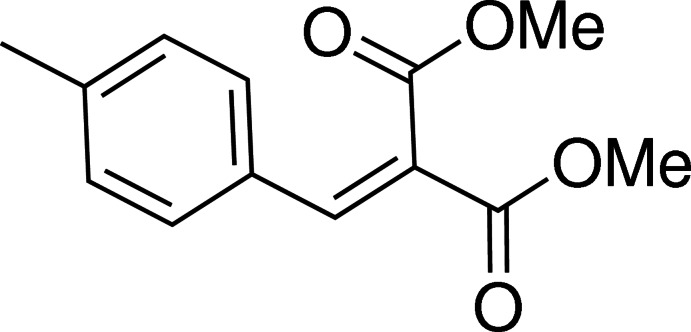


## Experimental

### 

#### Crystal data

C_13_H_14_O_4_
*M* *_r_* = 234.24
Monoclinic, 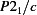

*a* = 14.0516 (6) Å
*b* = 7.7446 (3) Å
*c* = 12.5113 (5) Å
β = 113.727 (1)°
*V* = 1246.44 (9) Å^3^
*Z* = 4
Mo *K*α radiation
μ = 0.09 mm^−1^
*T* = 273 K
0.55 × 0.36 × 0.16 mm

#### Data collection

Bruker SMART APEX CCD area-detector diffractometer
Absorption correction: multi-scan (*SADABS*; Bruker, 2000 ▶) *T*_min_ = 0.951, *T*_max_ = 0.985
7125 measured reflections
2316 independent reflections
1850 reflections with *I* > 2σ(*I*)
*R*_int_ = 0.021

#### Refinement

*R*[*F* ^2^ > 2σ(*F* ^2^)] = 0.045
*wR*(*F* ^2^) = 0.136
*S* = 1.08
2316 reflections
154 parameters
H-atom parameters constrained
Δρ_max_ = 0.20 e Å^−3^
Δρ_min_ = −0.17 e Å^−3^

### 

Data collection: *SMART* (Bruker, 2000 ▶); cell refinement: *SAINT* (Bruker, 2000 ▶); data reduction: *SAINT*; program(s) used to solve structure: *SHELXS97* (Sheldrick, 2008 ▶); program(s) used to refine structure: *SHELXL97* (Sheldrick, 2008 ▶); molecular graphics: *SHELXTL* (Sheldrick, 2008 ▶); software used to prepare material for publication: *SHELXTL*, *PARST* (Nardelli, 1995 ▶) and *PLATON* (Spek, 2009 ▶).

## Supplementary Material

Crystal structure: contains datablock(s) global, I. DOI: 10.1107/S1600536813012464/rz5063sup1.cif

Structure factors: contains datablock(s) I. DOI: 10.1107/S1600536813012464/rz5063Isup2.hkl

Supplementary material file. DOI: 10.1107/S1600536813012464/rz5063Isup3.cml

Additional supplementary materials:  crystallographic information; 3D view; checkCIF report

## Figures and Tables

**Table 1:** Hydrogen-bond geometry (Å, °)

*D*—H⋯*A*	*D*—H	H⋯*A*	*D*⋯*A*	*D*—H⋯*A*
C13—H13*C*⋯O1^i^	0.96	2.49	3.442 (3)	170

Symmetry code: (i) 
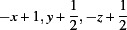
.

**Fig. 1.**
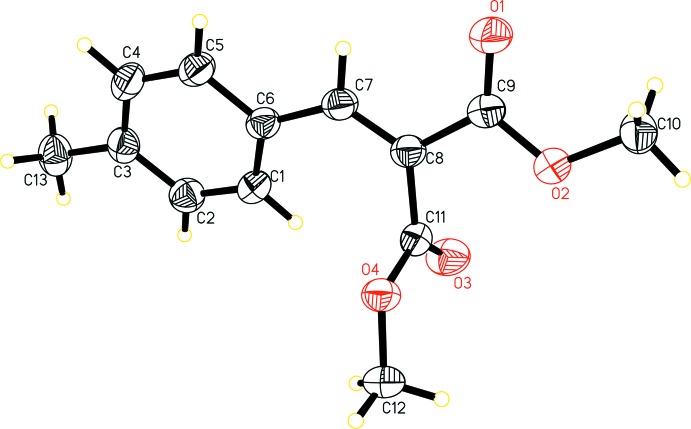
The molecular structure of title compound with displacement ellipsoids drawn at 30% probability level.

**Fig. 2.**
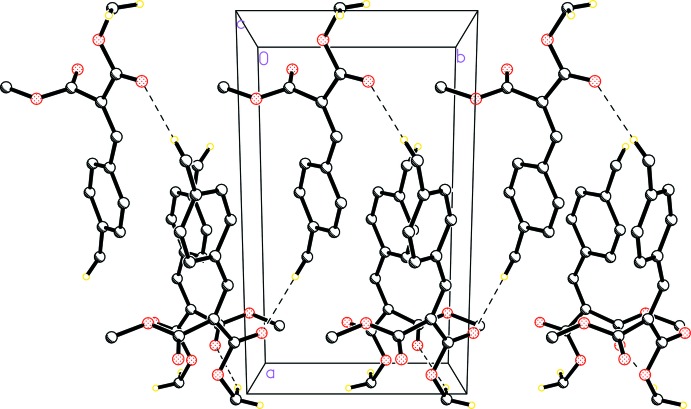
Crystal packing of the title compound viewed down the c axis. Only hydrogen atoms involved in hydrogen bonding (dashed lines) are shown.

## supplementary crystallographic information

### Comment

Alkylidene and arylidene malonates have attracted the attention of organic and medicinal chemists as building blocks of many organic compounds with diverse biological activities. Due to their distinct structural features, these compounds serve as precursors for Michael addition in multiple reactions, such as Aza-Michael addition, Mukaiyama-Michael reaction and Friedel-Crafts reactions (Liu *et al.*, 2012; Heydri & Tahamipour, 2011; Xu & Wang, 2011; Li *et al.*, 2010; Gallier *et al.*, 2009). Particularly they are utilized for the synthesis of trisubstituted alkenes *via* Knoevenagel condensation (Li *et al.*, 2011). These trisubstitued alkenes in turn can be useful for the preparation of various biologically active molecules (Besavaiah *et al.*, 2004).

The structure of title compound, C_13_H_14_O_4_, is composed of a dimethyl malonate (O1–O4/C8–C12) substituted benzylidene ring (C1–C7) (Fig. 1). The benzene ring forms dihedral angles of 18.60 (7) and 81.36 (8)° with the C9/C10/O1/O2 and C11/C12/O3/O4 side chains of malonate. In the crystal, the structure is stabilized *via* C13—H13C···O1 intermolecular interactions (Table 1) forming chains running parallel to the *b* axis (Fig. 2). All bond lengths and angles were found to be similar to those observed in other structurally related compounds (Rappoport & Gazit 1986).

### Experimental

To a 150 ml flame-dried round-bottom flask, equipped with a magnetic stir bar and fitted with a Dean-Stark apparatus, was added benzene (25 ml), toluene aldehyde (1.44 g, 12 mmol), piperidine (11 µ*L*, 0.12 mmol), acetic acid (7 µ*L*, 0.12 mmol), and dimethyl malonate (1.72 g, 13 mmol) under an argon atmosphere. The reaction mixture was allowed to reflux for 24 h. The reaction progress was monitored by ^1^H-NMR spectroscopy. After completion of the reaction, the reaction mixture was diluted with ethyl acetate (50 ml) and extracted with water (2 × 25 ml) and brine (1 × 25 ml) and dried over Na_2_SO_4_ to obtain the crude alkylidene malonate. Flash column chromatography (petroleum ether/ethyl acetate, 95:5 *v*/*v*) afforded a solution of the title compound as a clear liquid. On standing for 2 days at room temperature, cube-like crystals (2.67 g, 11.4 mmol, 95° yield) were obtained. M. p. 331 K. All chemicals were purchased from Sigma- Aldrich.

### Refinement

H atoms were positioned geometrically with C—H = 0.93–0.96 Å and constrained to ride on their parent atoms with *U*_iso_(H)= 1.2*U*_eq_ (C) or 1.5*U*_eq_ (C) for methyl H atoms.

### Crystal data

**Table:**

C_13_H_14_O_4_	*F*(000) = 496
*M**_r_* = 234.24	*D*_x_ = 1.248 Mg m^−^^3^
Monoclinic, *P*2_1_/*c*	Mo *K*α radiation, λ = 0.71073 Å
Hall symbol: -P 2ybc	Cell parameters from 2372 reflections
*a* = 14.0516 (6) Å	θ = 3.1–26.3°
*b* = 7.7446 (3) Å	µ = 0.09 mm^−^^1^
*c* = 12.5113 (5) Å	*T* = 273 K
β = 113.727 (1)°	Block, colourless
*V* = 1246.44 (9) Å^3^	0.55 × 0.36 × 0.16 mm
*Z* = 4	

### Data collection

**Table:**

Bruker SMART APEX CCD area-detector diffractometer	2316 independent reflections
Radiation source: fine-focus sealed tube	1850 reflections with *I* > 2σ(*I*)
Graphite monochromator	*R*_int_ = 0.021
ω scan	θ_max_ = 25.5°, θ_min_ = 3.1°
Absorption correction: multi-scan (*SADABS*; Bruker, 2000)	*h* = −17→11
*T*_min_ = 0.951, *T*_max_ = 0.985	*k* = −9→9
7125 measured reflections	*l* = −15→15

### Refinement

**Table:**

Refinement on *F*^2^	Primary atom site location: structure-invariant direct methods
Least-squares matrix: full	Secondary atom site location: difference Fourier map
*R*[*F*^2^ > 2σ(*F*^2^)] = 0.045	Hydrogen site location: inferred from neighbouring sites
*wR*(*F*^2^) = 0.136	H-atom parameters constrained
*S* = 1.08	*w* = 1/[σ^2^(*F*_o_^2^) + (0.0674*P*)^2^ + 0.2125*P*] where *P* = (*F*_o_^2^ + 2*F*_c_^2^)/3
2316 reflections	(Δ/σ)_max_ < 0.001
154 parameters	Δρ_max_ = 0.20 e Å^−^^3^
0 restraints	Δρ_min_ = −0.17 e Å^−^^3^

### Special details

**Table:**

Geometry. All e.s.d.'s (except the e.s.d. in the dihedral angle between two l.s. planes) are estimated using the full covariance matrix. The cell e.s.d.'s are taken into account individually in the estimation of e.s.d.'s in distances, angles and torsion angles; correlations between e.s.d.'s in cell parameters are only used when they are defined by crystal symmetry. An approximate (isotropic) treatment of cell e.s.d.'s is used for estimating e.s.d.'s involving l.s. planes.
Refinement. Refinement of *F*^2^ against ALL reflections. The weighted *R*-factor *wR* and goodness of fit *S* are based on *F*^2^, conventional *R*-factors *R* are based on *F*, with *F* set to zero for negative *F*^2^. The threshold expression of *F*^2^ > σ(*F*^2^) is used only for calculating *R*-factors(gt) *etc*. and is not relevant to the choice of reflections for refinement. *R*-factors based on *F*^2^ are statistically about twice as large as those based on *F*, and *R*- factors based on ALL data will be even larger.

### Fractional atomic coordinates and isotropic or equivalent isotropic displacement parameters (Å^2^)

**Table:**

	*x*	*y*	*z*	*U*_iso_*/*U*_eq_	
O1	0.15387 (12)	−0.05063 (19)	0.26275 (14)	0.0835 (5)	
O2	0.03912 (10)	0.15029 (16)	0.16332 (11)	0.0631 (4)	
O3	0.10137 (11)	0.30017 (18)	−0.02578 (11)	0.0775 (5)	
O4	0.19102 (10)	0.45753 (15)	0.13086 (11)	0.0621 (4)	
C1	0.36456 (14)	0.2423 (2)	0.04658 (15)	0.0594 (5)	
H1A	0.2996	0.2897	0.0027	0.071*	
C2	0.44558 (15)	0.2730 (2)	0.01473 (16)	0.0635 (5)	
H2A	0.4340	0.3405	−0.0508	0.076*	
C3	0.54418 (14)	0.2067 (2)	0.07690 (16)	0.0589 (5)	
C4	0.55784 (15)	0.1057 (3)	0.17338 (17)	0.0649 (5)	
H4A	0.6230	0.0592	0.2172	0.078*	
C5	0.47681 (15)	0.0728 (2)	0.20574 (16)	0.0607 (5)	
H5A	0.4883	0.0034	0.2704	0.073*	
C6	0.37811 (13)	0.1409 (2)	0.14407 (14)	0.0521 (4)	
C7	0.29555 (14)	0.0931 (2)	0.18075 (14)	0.0540 (4)	
H7A	0.3116	0.0001	0.2321	0.065*	
C8	0.20104 (13)	0.1592 (2)	0.15361 (14)	0.0517 (4)	
C9	0.13063 (14)	0.0737 (2)	0.20022 (15)	0.0555 (4)	
C10	−0.03757 (16)	0.0739 (3)	0.19883 (19)	0.0683 (5)	
H10A	−0.1005	0.1405	0.1677	0.103*	
H10B	−0.0114	0.0726	0.2825	0.103*	
H10C	−0.0517	−0.0422	0.1698	0.103*	
C11	0.15819 (13)	0.3100 (2)	0.07484 (14)	0.0512 (4)	
C12	0.15580 (17)	0.6132 (2)	0.0614 (2)	0.0775 (6)	
H12A	0.1839	0.7125	0.1098	0.116*	
H12B	0.0813	0.6181	0.0295	0.116*	
H12C	0.1790	0.6120	−0.0011	0.116*	
C13	0.63181 (16)	0.2386 (3)	0.03988 (19)	0.0766 (6)	
H13A	0.6081	0.3113	−0.0280	0.115*	
H13B	0.6554	0.1305	0.0219	0.115*	
H13C	0.6880	0.2943	0.1021	0.115*	

### Atomic displacement parameters (Å^2^)

**Table:**

	*U*^11^	*U*^22^	*U*^33^	*U*^12^	*U*^13^	*U*^23^
O1	0.0768 (10)	0.0778 (10)	0.1055 (11)	0.0191 (8)	0.0467 (8)	0.0389 (8)
O2	0.0565 (8)	0.0567 (7)	0.0771 (8)	0.0059 (6)	0.0280 (6)	0.0078 (6)
O3	0.0810 (10)	0.0704 (9)	0.0579 (8)	0.0043 (7)	0.0039 (7)	0.0042 (6)
O4	0.0622 (8)	0.0465 (7)	0.0703 (8)	0.0023 (6)	0.0191 (6)	0.0000 (5)
C1	0.0510 (10)	0.0607 (11)	0.0617 (10)	0.0083 (8)	0.0176 (8)	0.0066 (8)
C2	0.0654 (12)	0.0638 (12)	0.0616 (10)	0.0018 (9)	0.0260 (9)	0.0032 (9)
C3	0.0556 (10)	0.0563 (10)	0.0629 (10)	−0.0051 (8)	0.0218 (8)	−0.0171 (8)
C4	0.0476 (10)	0.0688 (12)	0.0669 (11)	0.0076 (9)	0.0111 (8)	−0.0072 (9)
C5	0.0561 (11)	0.0598 (11)	0.0582 (10)	0.0083 (9)	0.0148 (8)	0.0043 (8)
C6	0.0517 (10)	0.0460 (9)	0.0531 (9)	0.0034 (7)	0.0154 (7)	−0.0030 (7)
C7	0.0561 (11)	0.0482 (9)	0.0529 (9)	0.0039 (8)	0.0169 (8)	0.0051 (7)
C8	0.0525 (10)	0.0454 (9)	0.0524 (9)	0.0017 (7)	0.0161 (7)	−0.0002 (7)
C9	0.0584 (11)	0.0488 (10)	0.0579 (10)	0.0051 (8)	0.0220 (8)	0.0023 (8)
C10	0.0605 (11)	0.0678 (12)	0.0826 (13)	−0.0028 (10)	0.0349 (10)	−0.0023 (10)
C11	0.0438 (9)	0.0518 (10)	0.0551 (9)	0.0020 (7)	0.0170 (7)	0.0010 (7)
C12	0.0783 (14)	0.0483 (11)	0.1068 (16)	0.0099 (10)	0.0383 (13)	0.0147 (10)
C13	0.0645 (12)	0.0831 (14)	0.0874 (14)	−0.0078 (11)	0.0359 (11)	−0.0216 (11)

### Geometric parameters (Å, º)

**Table:**

O1—C9	1.200 (2)	C5—H5A	0.9300
O2—C9	1.319 (2)	C6—C7	1.457 (2)
O2—C10	1.447 (2)	C7—C8	1.334 (2)
O3—C11	1.1913 (19)	C7—H7A	0.9300
O4—C11	1.3226 (19)	C8—C11	1.490 (2)
O4—C12	1.452 (2)	C8—C9	1.491 (2)
C1—C2	1.370 (3)	C10—H10A	0.9600
C1—C6	1.398 (2)	C10—H10B	0.9600
C1—H1A	0.9300	C10—H10C	0.9600
C2—C3	1.386 (3)	C12—H12A	0.9600
C2—H2A	0.9300	C12—H12B	0.9600
C3—C4	1.385 (3)	C12—H12C	0.9600
C3—C13	1.500 (3)	C13—H13A	0.9600
C4—C5	1.377 (3)	C13—H13B	0.9600
C4—H4A	0.9300	C13—H13C	0.9600
C5—C6	1.391 (2)		
			
C9—O2—C10	116.73 (15)	C11—C8—C9	116.87 (15)
C11—O4—C12	115.97 (14)	O1—C9—O2	124.07 (17)
C2—C1—C6	120.92 (16)	O1—C9—C8	124.21 (16)
C2—C1—H1A	119.5	O2—C9—C8	111.71 (15)
C6—C1—H1A	119.5	O2—C10—H10A	109.5
C1—C2—C3	122.19 (18)	O2—C10—H10B	109.5
C1—C2—H2A	118.9	H10A—C10—H10B	109.5
C3—C2—H2A	118.9	O2—C10—H10C	109.5
C4—C3—C2	117.06 (18)	H10A—C10—H10C	109.5
C4—C3—C13	121.26 (18)	H10B—C10—H10C	109.5
C2—C3—C13	121.67 (18)	O3—C11—O4	123.90 (16)
C5—C4—C3	121.31 (17)	O3—C11—C8	124.73 (16)
C5—C4—H4A	119.3	O4—C11—C8	111.37 (14)
C3—C4—H4A	119.3	O4—C12—H12A	109.5
C4—C5—C6	121.63 (18)	O4—C12—H12B	109.5
C4—C5—H5A	119.2	H12A—C12—H12B	109.5
C6—C5—H5A	119.2	O4—C12—H12C	109.5
C5—C6—C1	116.89 (17)	H12A—C12—H12C	109.5
C5—C6—C7	118.11 (16)	H12B—C12—H12C	109.5
C1—C6—C7	124.87 (15)	C3—C13—H13A	109.5
C8—C7—C6	131.27 (16)	C3—C13—H13B	109.5
C8—C7—H7A	114.4	H13A—C13—H13B	109.5
C6—C7—H7A	114.4	C3—C13—H13C	109.5
C7—C8—C11	124.44 (16)	H13A—C13—H13C	109.5
C7—C8—C9	118.63 (15)	H13B—C13—H13C	109.5
			
C6—C1—C2—C3	−0.3 (3)	C6—C7—C8—C9	176.17 (16)
C1—C2—C3—C4	0.5 (3)	C10—O2—C9—O1	−2.0 (3)
C1—C2—C3—C13	178.91 (17)	C10—O2—C9—C8	177.38 (14)
C2—C3—C4—C5	0.1 (3)	C7—C8—C9—O1	1.2 (3)
C13—C3—C4—C5	−178.38 (17)	C11—C8—C9—O1	178.61 (17)
C3—C4—C5—C6	−0.8 (3)	C7—C8—C9—O2	−178.12 (15)
C4—C5—C6—C1	0.9 (3)	C11—C8—C9—O2	−0.7 (2)
C4—C5—C6—C7	177.10 (16)	C12—O4—C11—O3	−1.6 (3)
C2—C1—C6—C5	−0.4 (3)	C12—O4—C11—C8	178.58 (14)
C2—C1—C6—C7	−176.28 (17)	C7—C8—C11—O3	98.3 (2)
C5—C6—C7—C8	166.38 (18)	C9—C8—C11—O3	−78.9 (2)
C1—C6—C7—C8	−17.8 (3)	C7—C8—C11—O4	−81.9 (2)
C6—C7—C8—C11	−1.0 (3)	C9—C8—C11—O4	100.88 (17)

### Hydrogen-bond geometry (Å, º)

**Table:**

*D*—H···*A*	*D*—H	H···*A*	*D*···*A*	*D*—H···*A*
C13—H13*C*···O1^i^	0.96	2.49	3.442 (3)	170

Symmetry code: (i) −*x*+1, *y*+1/2, −*z*+1/2.
